# Interactive Multimedia to Teach the Life Cycle of *Trypanosoma cruzi*, the Causative Agent of Chagas Disease

**DOI:** 10.1371/journal.pntd.0001749

**Published:** 2012-08-28

**Authors:** Dirceu E. Teixeira, Marlene Benchimol, Paulo Henrique Crepaldi, Wanderley de Souza

**Affiliations:** 1 Research Department, CEDERJ Consortium/CECIERJ Foundation, Rio de Janeiro, RJ, Brazil; 2 National Institute of Metrology, Quality and Technology (INMETRO), Rio de Janeiro, RJ, Brazil; 3 Santa Úrsula University, Rio de Janeiro, Brazil; 4 Laboratory of Cellular Ultrastructure Hertha Meyer, Institute of Biophysics Carlos Chagas Filho, Federal University of Rio de Janeiro, Rio de Janeiro, RJ, Brazil; Instituto Oswaldo Cruz, Fiocruz, Brazil

## Introduction

Parasitic protozoa are important agents of human and veterinary diseases, which are widely distributed throughout the world. The parasite *Trypanosoma cruzi*, which is the causal agent of the human disease known as Chagas disease, affects approximately 8 million people and causes more than 14,000 deaths per year in Latin America. It is estimated that in Brazil there are around 2 million individuals infected [1]. *T. cruzi* has a complex life cycle involving both vertebrate and invertebrate hosts in three well-defined developmental stages: (1) amastigotes, which are the proliferative forms found inside the vertebrate host cells; (2) epimastigotes, which are the proliferative forms found in the intestine of the invertebrate host; and (3) trypomastigotes, which are highly infective and originate from the amastigotes at the end of the intracellular cycle following their release into the intercellular space and into bloodstream [2]. Trypomastigotes also arise from epimastigotes in the posterior regions of the digestive tract of the invertebrate host [3].

The present work aims to use a cell biologic approach to create multimedia materials that present basic aspects of the life cycle of *T. cruzi* and the morphology of its various developmental stages, as well as some biological processes such a division, motility, and endocytic activity.

The current teaching method is based upon formal lectures using classic material with little emphasis on the use of three-dimensional (3D) animation models. In this report, we present new instructional material with modern schemes and dynamic models that include 3D animations (Box 1). These educational tools will be useful for a broad audience, which includes students in face-to-face and distance education, teachers, researchers, and any member of the general public that are interested in parasites. As an instructional tool, the animations are more effective than the static graphics for teaching dynamic events [4]. Studies in biology courses have shown that animations lead to increased student understanding and retention of cell biology information [5].

**Box 1. Advantages and Disadvantages of Scientific Animation**

**Advantages**

1. Animation is a powerful tool to communicate abstract scientific ideas that are difficult to visualize and interpret when described with words or using static images
2. Increase student understanding and memory retention
3. Animations are playful and accessible to undergraduate students and enable them to understand complex processes more easily

**Disadvantages**

1. High cost
2. Require a great deal of time
3. Team and software specialized

## Methods

The 3D models and animations were produced by designers working at the CECIERJ Foundation (Fundação Centro de Ciências e Educação Superior a Distância do Estado do Rio de Janeiro - CEDERJ Consortium).

Our analysis is based on information obtained by our group in the last 20 years using video microscopy and light microscopy as well as scanning and transmission electron microscopy, which show various aspects of the structural organization of the protozoan and its interaction with host cells. Our analysis also used information obtained by different research groups. All animations and images were produced using software such as 3ds Max, Maya, Poser, and Flash.

## Results and Discussion

### Life Cycle

**Figure 1:**
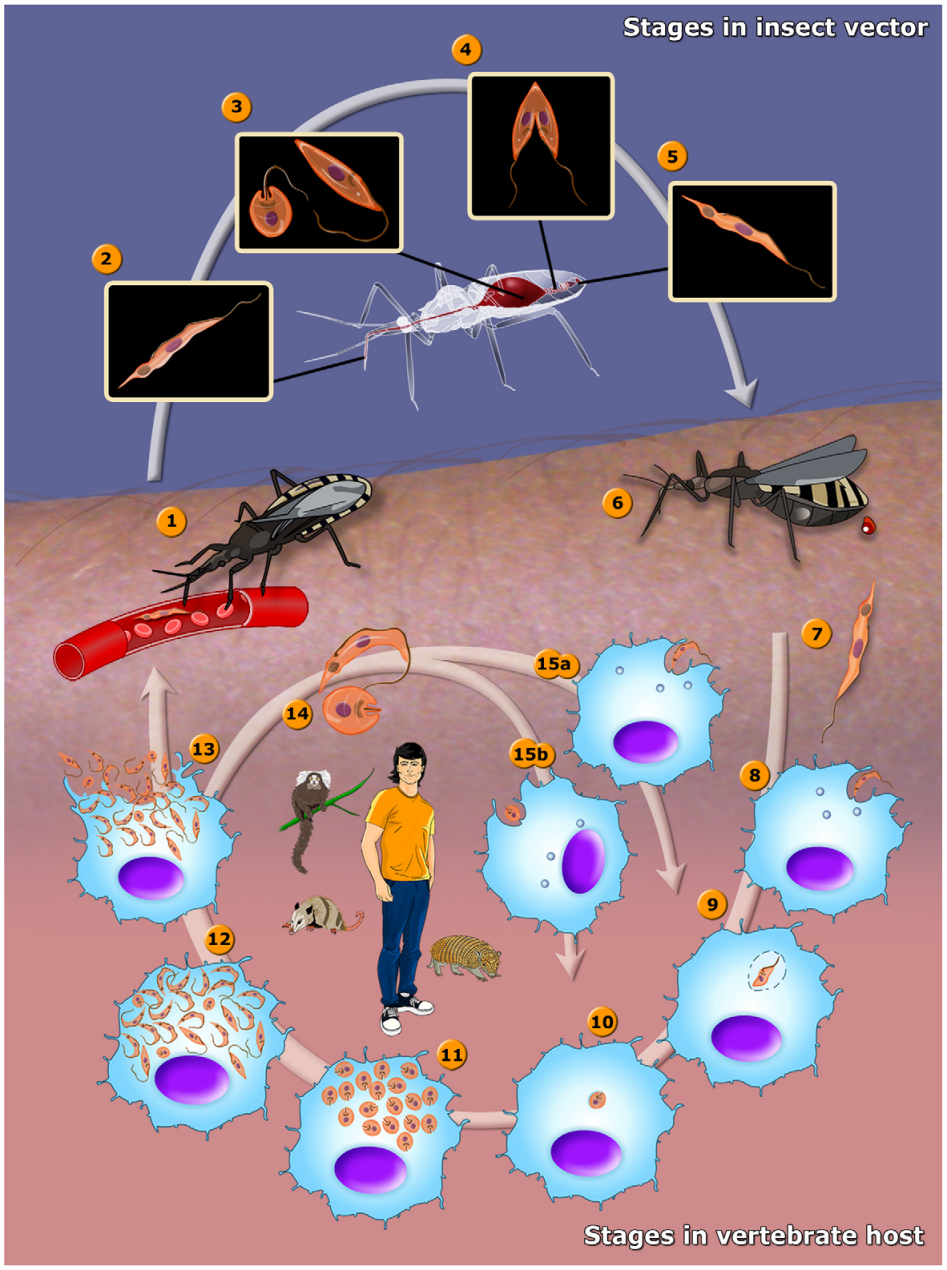
The life cycle of *T. cruzi*. 1. The insect vector (female or male) bites a mammalian host and ingests trypomastigotes located in the blood. 2. Metacyclic trypomastigotes. 3. Trypomastigotes transform into epimastigotes and some spheromastigotes. 4. Epimastigotes multiply in the midgut. 5. Epimastigotes transform into metacyclic trypomastigotes in the hindgut. 6. The insect vector passes the metacyclic trypomastigotes in feces near a bite site after feeding on a mammalian host. 7. Metacyclic trypomastigotes form. 8. Metacyclic trypomastigote infects macrophages. 9. Metacyclic trypomastigote transforms into amastigote. 10. Amastigote is released from the parasitophorous vacuole. 11. Amastigotes multiply in the cytoplasm. 12. Amastigotes transform into trypomastigotes. 13. Trypomastigotes burst out of the cell. 14. Amastigotes and trypomastigotes form. 15. (a) Trypomastigotes and (b) amastigotes infect macrophages. In the central portion of the figure, we added the most important animal reservoirs involved in the maintenance of the parasite in the domestic and peridomestic environment.

During its life cycle, *T. cruzi* infects both invertebrate and vertebrate hosts. Figure 1 shows a general view of its life cycle of the basic aspects of the life cycle of *T. cruzi* in the human host (video: http://www.imbebb.org.br/conteudo.asp?idsecao=242) and in the triatomine insect (video: http://www.imbebb.org.br/conteudo.asp?idsecao=243).

### Morphology of *T. cruzi*

**Figure 2:**
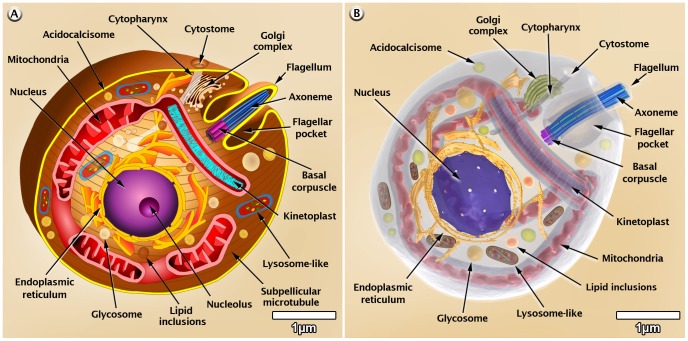
Schematic representations of *T. cruzi* amastigote organelles. (A) 2D and (B) 3D models. These images were made based on micrographs of light microscopy as well as scanning and transmission electron microscopy.

On the basis of several images obtained by scanning and transmission electron microscopy, we made 3D figures that illustrate the general shape of the various developmental stages of *T. cruzi* as well as the presence and distribution of structures and organelles, as shown in Figures 2–4. A more detailed 3D animation of the ultrastructure of each developmental stage is shown in the videos found at http://www.imbebb.org.br/conteudo.asp?idsecao=253, http://www.imbebb.org.br/conteudo.asp?idsecao=252, and http://www.imbebb.org.br/conteudo.asp?idsecao=251.

**Figure 3 pntd-0001749-g003:**
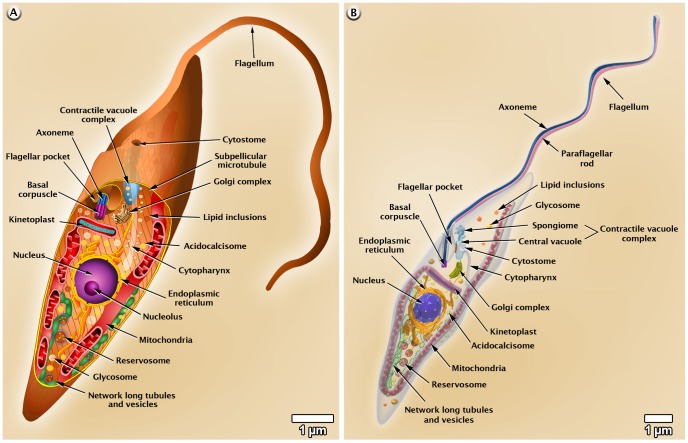
Schematic representations of *T. cruzi* epimastigote organelles. (A) 2D and (B) 3D models. These images were made based on micrographs of light microscopy as well as scanning and transmission electron microscopy.

**Figure 4:**
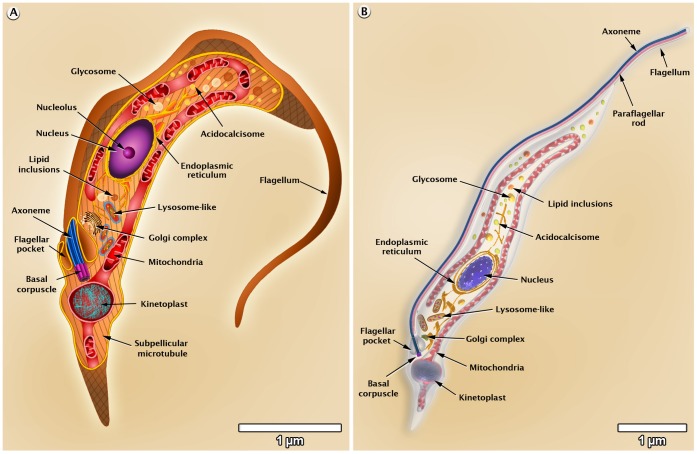
Schematic representations of *T. cruzi* trypomastigote organelles. (A) 2D and (B) 3D models. These images were made based on micrographs of light microscopy as well as scanning and transmission electron microscopy.

### Dynamic Processes

**Figure 5:**
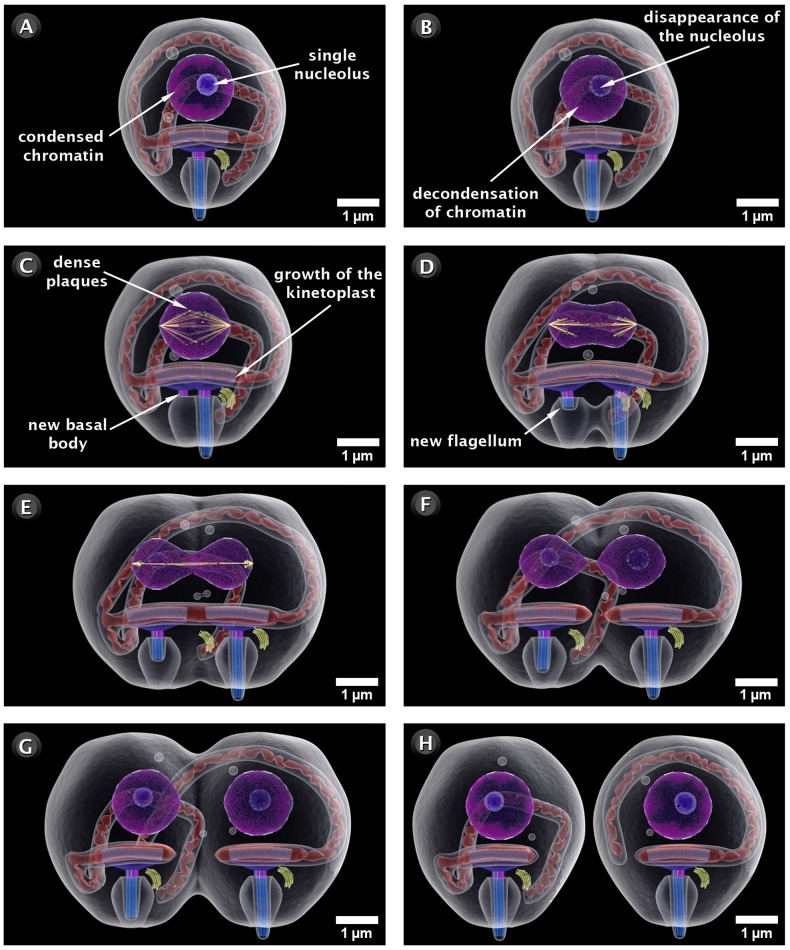
General view depicting the stages of amastigote division by binary fission. (A) In the preliminary phase of cell division, the nucleus displays condensed chromatin and a single nucleolus. (B) The early phase of the division process begins with the decondensation of chromatin and the disappearance of the nucleolus. (C) In the equatorial stage, there is early lateral growth of the kinetoplast and the appearance of a new basal body. This change is followed by the appearance of an arranged set of ten dense plaques in the equatorial region of the nucleus. These plaques are associated with an intranuclear spindle formed by microtubules. (D) The early elongational phase begins with the splitting of dense plaques, which migrate toward the nucleus poles. The nucleus elongates, and the spindle microtubules modify their distribution. The new flagellum emerges from the flagellar pocket. (E) In the final phase of elongation, the split dense plaques begin to migrate toward the nucleus poles, which exhibit an hourglass shape. This form represents the last stage of nucleus constriction. (F) In the reorganizative phase, the microtubules fade out in a stepwise fashion, the nucleolus begins to reconstitute, and the chromatin begins to condense. In this phase, the nuclei and kinetoplasts are already individualized. (G) At the stage of constriction, cytokinesis occurs and culminates with the formation of two independent amastigotes (H). These images were made based on micrographs of transmission electron microscopy.

**Figure 6:**
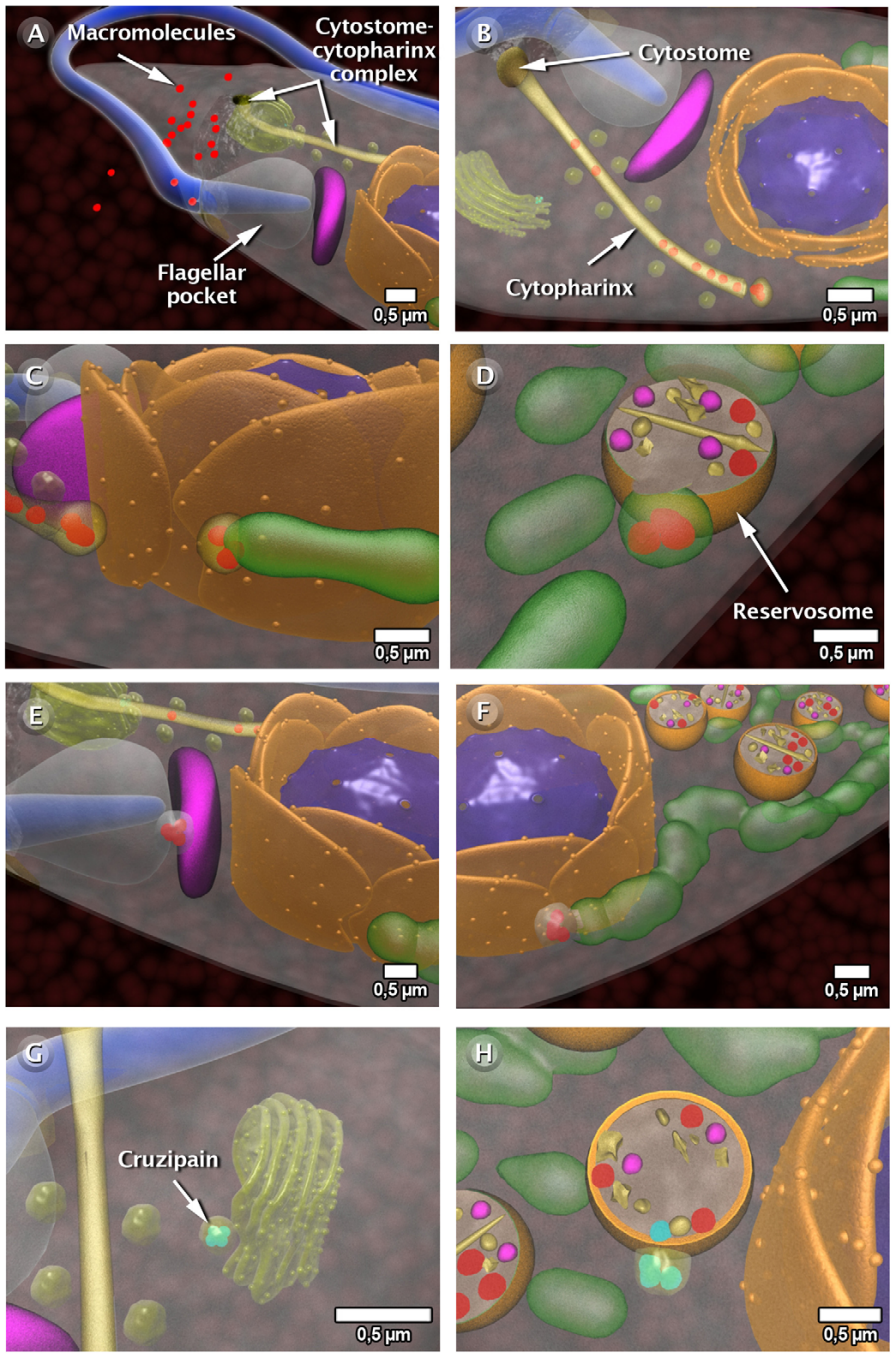
The endocytic pathway in the epimastigote form of (A) Endocytosis occurs in two sites of macromolecular ingestion: the cytostome-cytopharinx complex and the flagellar pocket. (B) In cytostome-cytopharinx complex the macromolecules migrate through the cytopharynx and are internalized via small vesicles, which are formed in the final portion of the cytopharynx. (C) Subsequently, the macromolecules cross through the early tubular endosomal network and are delivered to a reservosome (D). (E) The macromolecules are also internalized via vesicles that form in the flagellar pocket. (F) The endocytic pathway continues through a network of long tubules and vesicles extending to the posterior end of the cell body, returning to the opposite direction and eventually merging with the reservosome. (G) Our model also suggests that cruzipain molecules, as well as other proteases, are processed and leave the Golgi complex. (H) Vesicles containing these molecules also interact with the endocytic pathway and are transported to reservosomes. These images were made based on micrographs of transmission electron microscopy.

**Figure 7:**
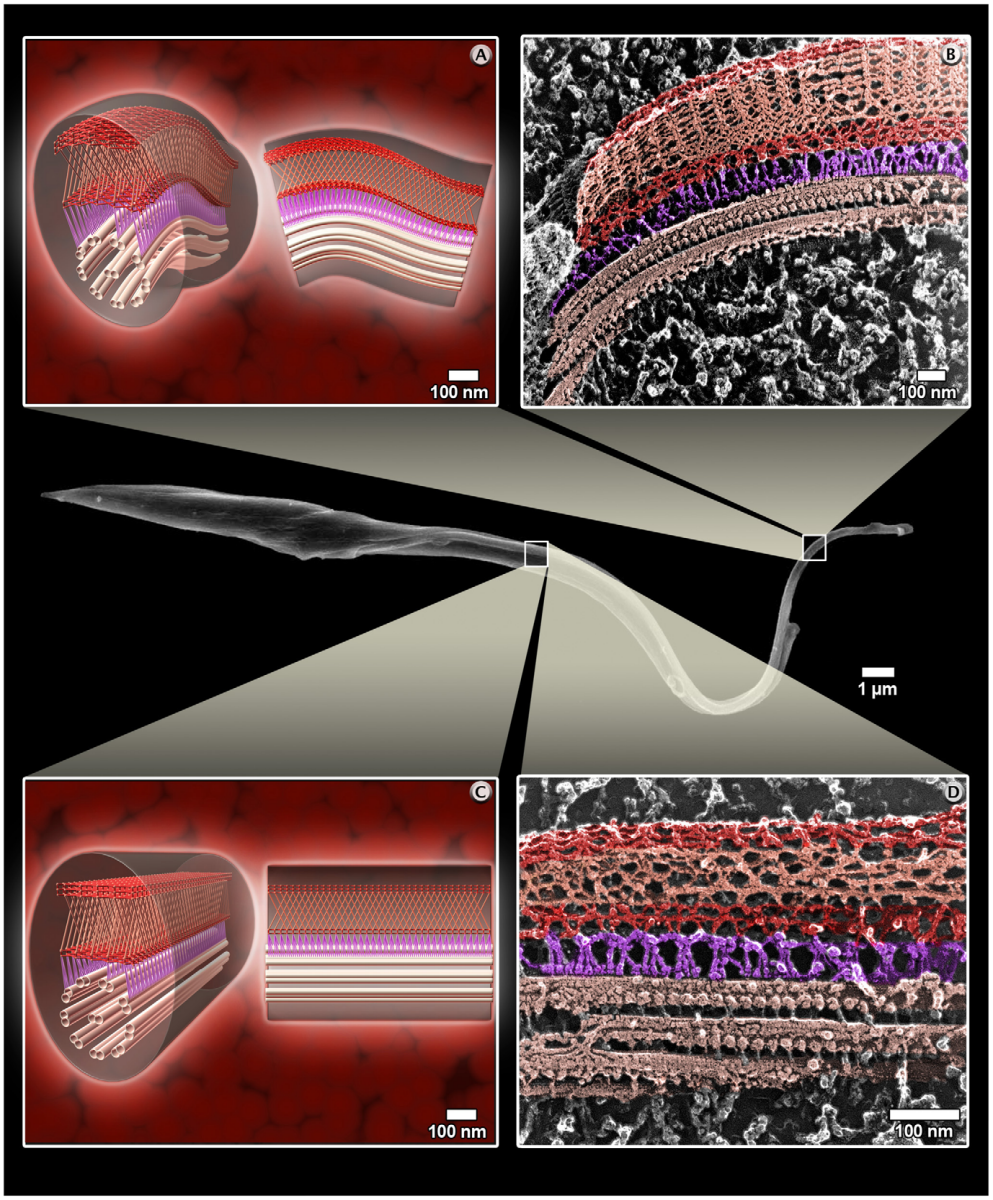
Frame view of paraflagellar rod animation during flagellar beating in comparison to deep-etching replicas. (A and B) Show the flagellum in a straight state and (C and D) in a bent state. (A and C) Schematic 3D representation and (B and D) deep-etching replica images. Axoneme (light pink), filaments that link the PFR to the axoneme (purple), proximal and distal domains of the PFR (red), and the intermediate domain (salmon). These schematic 3D representations were made based on micrographs of transmission electron microscopy (image courtesy of Gustavo Rocha).

We analyzed some of the dynamic processes, which take place in the *T. cruzi* cell cycle as (a) cell division (Figure 5 and http://www.imbebb.org.br/conteudo.asp?idsecao=250), (b) the highly polarized endocytic activity where the epimastigote forms uptake macromolecules from the medium as previously discussed in a recent review [6] (Figure 6 and http://www.imbebb.org.br/conteudo.asp?idsecao=249), and (c) the structural organization of the paraflagellar rod (PFR), which is a structure closely associated to the axoneme and a component of the flagellum of most of the trypanosomatids [7], [8]. On the basis of the images obtained using atomic force microscopy (AFM) and transmission electron microscopy of freeze-fractured and deep-etched cells, we were able to propose a model for the PFR [9]. A schematic motility is represented as a 3D hypothesis of the PFR (Figure 7 and video: http://www.imbebb.org.br/conteudo.asp?idsecao=248).

### The Behavior of *T. cruzi* in the Invertebrate Host

**Figure 8:**
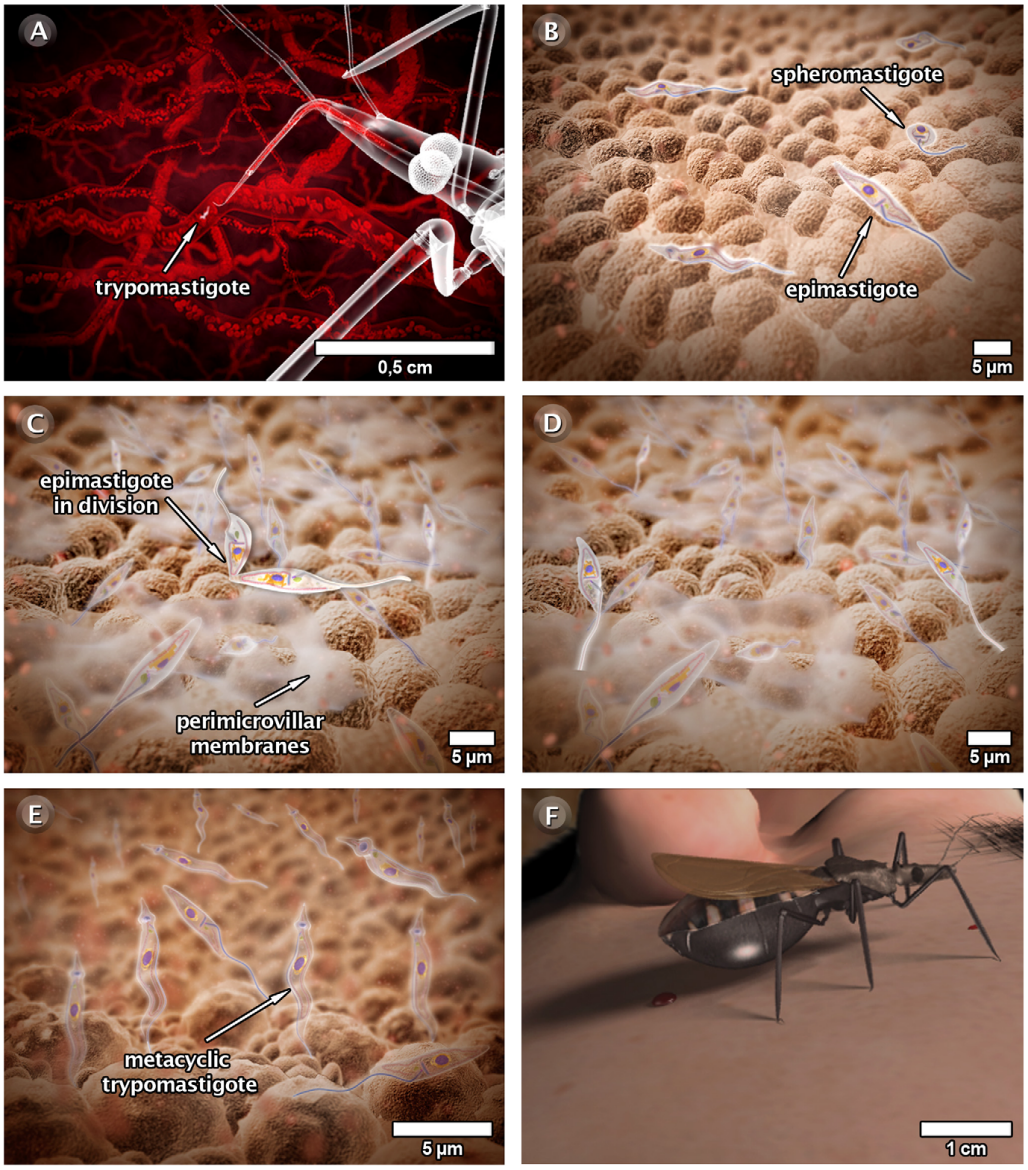
Schematic 3D view of the phases of *T. cruzi* interaction in the invertebrate host. (A) Insect vector ingesting trypomastigotes present in the blood of the vertebrate host during a blood meal. (B) In the stomach of the insect, trypomastigotes transform into epimastigotes and spheromastigotes. (C) Epimastigotes multiply in the midgut and attach to the perimicrovillar membranes of the intestinal cells. (D) Note that this adhesion occurs predominantly through the region of the flagellum. (E) At the most posterior region, many of the epimastigotes transform into metacyclic trypomastigotes and adhere to the cuticle lining the epithelium of the rectum and the rectal sac of the insect. (F) When the parasites leave the epithelium, the metacyclic trypomastigotes may be eliminated in the urine or feces of the insect. These images were made based on micrographs of transmission electron microscopy and video microscopy.

Figure 8 and the video at http://www.imbebb.org.br/conteudo.asp?idsecao=243 show the life cycle of *T. cruzi* in the invertebrate host.

### The Interaction of *T. cruzi* with Vertebrate Host Cells

**Figure 9:**
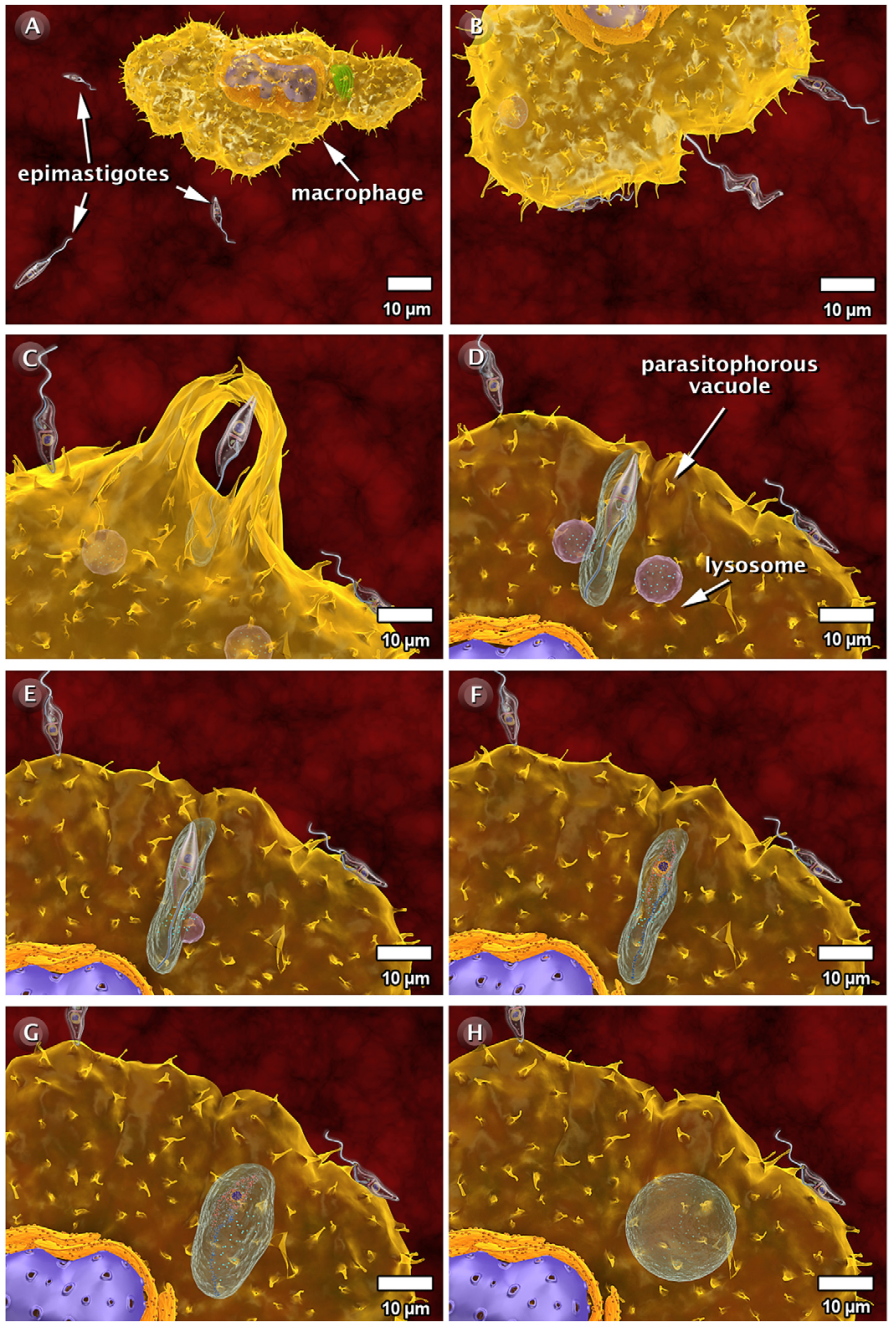
Schematic 3D view of the phases of interaction of the epimastigote form of *T. cruzi* with vertebrate cells (macrophage). (A) Attachment of epimastigotes to the macrophage surface. (B) This attachment triggers the internalization process via phagocytosis with the formation of pseudopods (C) and is followed by the formation of a parasitophorous vacuole. (D–G) Host cell lysosomes migrate toward and fuse with the parasitophorous vacuole, releasing their contents into the vacuole and subsequently digesting the intravacuolar epimastigotes (H). These images were made based on micrographs of transmission electron microscopy and video microscopy.

**Figure 10 pntd-0001749-g010:**
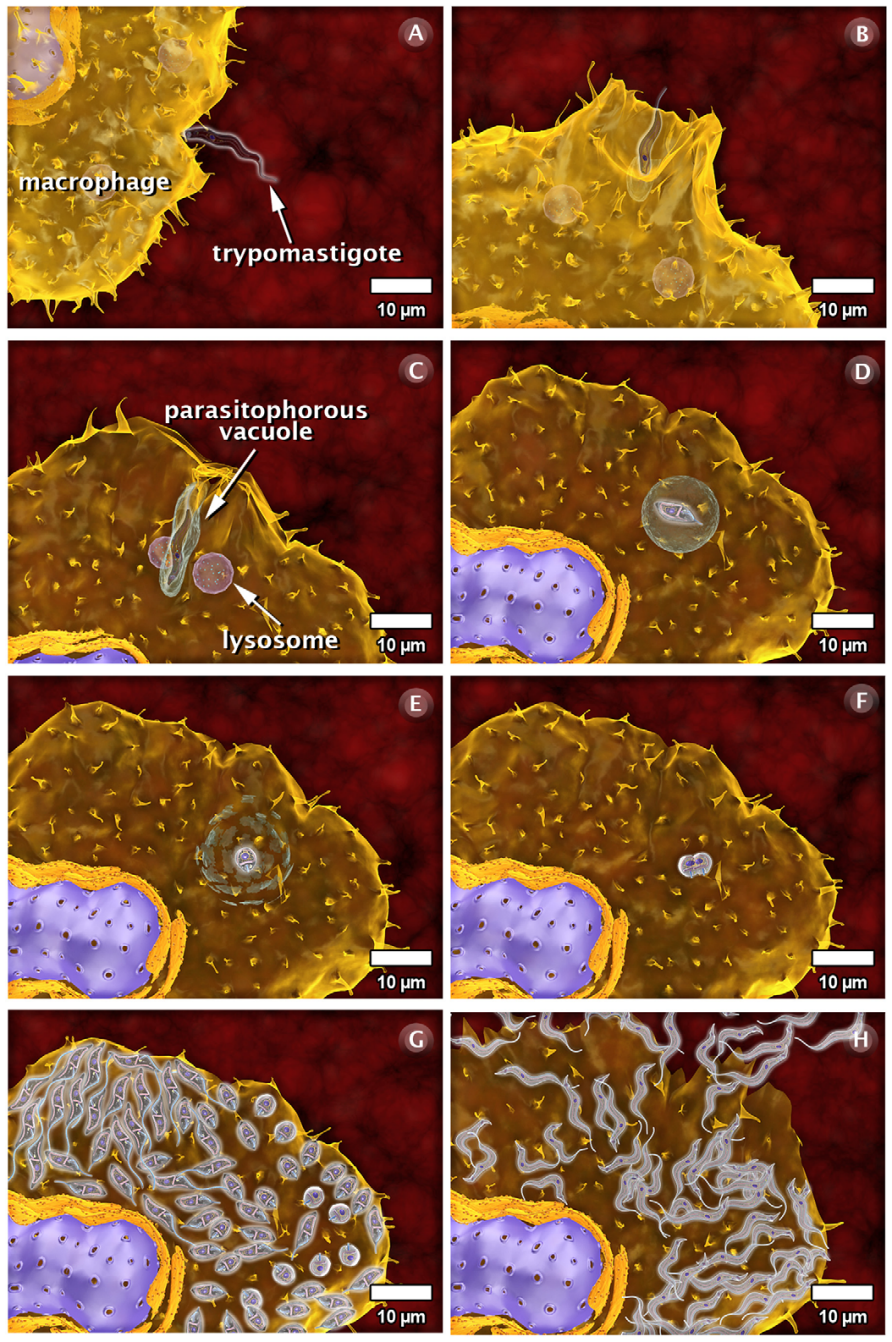
Schematic 3D view of the phases of interaction of the trypomastigote form of *T. cruzi* with vertebrate cells (macrophage). (A) Attachment of the trypomastigote form to the macrophage surface. (B) The process of internalization via phagocytosis begins with the formation of pseudopods and is followed by the recruitment and fusion of host cell lysosomes (C). A parasitophorous vacuole is subsequently formed. The lysosomal content is released into the vacuole, and the parasite is not affected. (D) In the vacuole, the trypomastigote transforms into the amastigote form. (E) This transformation is accompanied by the digestion of the parasitophorous vacuole membrane. (F) The amastigote is released into the cytoplasm of the host cell and divide several times. (G) Following division, the amastigotes transform into trypomastigotes, which show intense and constant movement. (H) The host cell bursts and the parasites reach the extracellular space and, subsequently, the bloodstream. These images were made based on micrographs of transmission electron microscopy and video microscopy.

**Figure 11:**
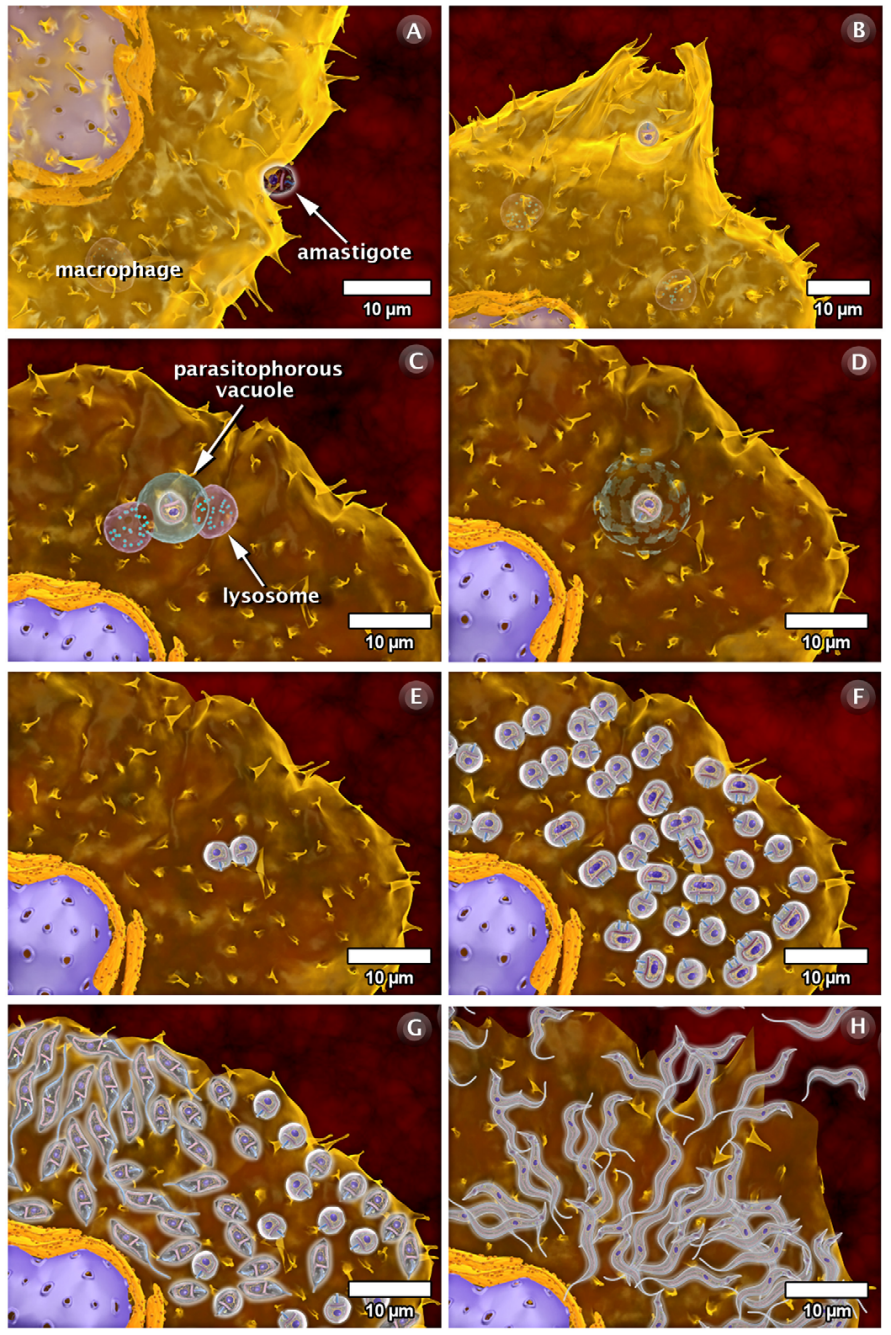
Schematic 3D view of the phases of interaction of the amastigote form of *T. cruzi* with vertebrate cells (macrophage). (A) In this example, attachment of the amastigote form to the macrophage surface is observed to initiate the process of internalization via phagocytosis. (B) The formation of pseudopods is followed by the formation of a parasitophorous vacuole (C). The lysosomes fuse with the parasitophorous vacuole and discharge their contents. (D) Subsequent digestion of the parasitophorous vacuole membrane occurs. (E) Note that the amastigote is released into the cytoplasm of the host cell and divides several times (F). (G) Following division, the amastigotes transform in trypomastigotes, which display intense and constant movement. (H) Finally, the host cell bursts and the parasites are released into the extracellular space and reach the bloodstream. These images were made based on micrographs of transmission electron microscopy and video microscopy.

**Figure 12:**
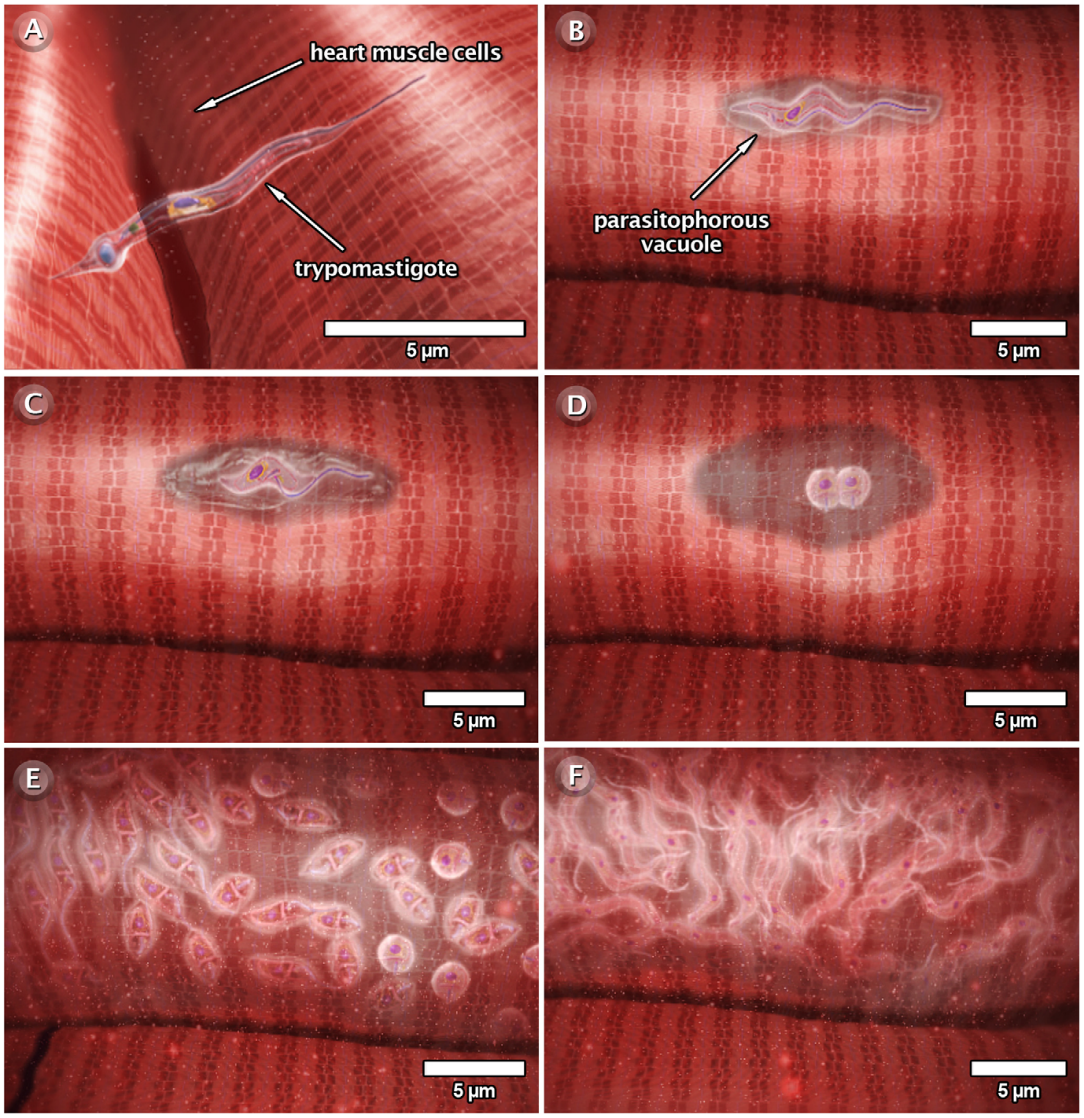
Schematic 3D view of the phases of interaction of the trypomastigote form of *T. cruzi* with vertebrate cells (cardiac cells). (A) Attachment of the trypomastigote form to the surface of heart muscle cells. This attachment initiates the process of invasion and is followed by the formation of a parasitophorous vacuole (B). (C) Inside the vacuole, the trypomastigote transforms into an amastigote form and this transformation is accompanied by the digestion of the parasitophorous vacuole membrane. (D) The amastigote is released into the cytoplasm of the host cell and divides several times (E). (F) Following division, the amastigotes transform into trypomastigotes, which are released into the extracellular space. These images were made based on micrographs of transmission electron microscopy and video microscopy.

As part of the life cycle, the infective trypomastigote and amastigote forms of *T. cruzi* interact with different types of cells in the mammalian hosts, such as macrophages, muscle cells, epithelial cells, and neurons. This interaction has been studied in some detail in cell culture (both phagocytic and non-professional phagocytic cells). Figures 9–11 and the next three videos illustrate the macrophage interactions with the non-infective epimastigotes as well as the infective amastigote and trypomastigote forms. For epimastigotes, the destruction of the intravacuolar parasite occurs (Figure 9, video: http://www.imbebb.org.br/conteudo.asp?idsecao=247). In trypomastigotes, fusion of the lysosomes with the parasitophorous vacuole (PV) occurs even during gradual transformation of trypomastigotes into amastigotes (Figure 10, video: http://www.imbebb.org.br/conteudo.asp?idsecao=246). A similar process occurs when amastigotes infect host cells (Figure 11, video: http://www.imbebb.org.br/conteudo.asp?idsecao=245). Figure 12 and the video at http://www.imbebb.org.br/conteudo.asp?idsecao=244 show the process of infection in heart muscle cells, where the intracellular cycle resembles that described for macrophages.

Taken together, the 3D schematics shown in Figures 9–11 and the dynamic 3D videos of interaction between the forms of *T. cruzi* and macrophage cells allow a better visualization of the various developmental stages of *T. cruzi*, including dynamic cellular processes as well as the interaction of the protozoan with vertebrate and invertebrate hosts. The multimedia materials described herein will present a comprehensive view of the protozoan life cycle to students. These materials also offer dynamic models that improve our understanding of some important biological processes.
